# Risk Prediction Model for Colorectal Cancer: National Health Insurance Corporation Study, Korea

**DOI:** 10.1371/journal.pone.0088079

**Published:** 2014-02-12

**Authors:** Aesun Shin, Jungnam Joo, Hye-Ryung Yang, Jeongin Bak, Yunjin Park, Jeongseon Kim, Jae Hwan Oh, Byung-Ho Nam

**Affiliations:** 1 Molecular Epidemiology Branch, National Cancer Center, Goyang-si, Republic of Korea; 2 Biometric Research Branch, National Cancer Center, Goyang-si, Republic of Korea; 3 Center for Colorectal Cancer, National Cancer Center Hospital, National Cancer Center, Goyang-si, Republic of Korea; Nanjing Medical University, China

## Abstract

**Purpose:**

Incidence and mortality rates of colorectal cancer have been rapidly increasing in Korea during last few decades. Development of risk prediction models for colorectal cancer in Korean men and women is urgently needed to enhance its prevention and early detection.

**Methods:**

Gender specific five-year risk prediction models were developed for overall colorectal cancer, proximal colon cancer, distal colon cancer, colon cancer and rectal cancer. The model was developed using data from a population of 846,559 men and 479,449 women who participated in health examinations by the National Health Insurance Corporation. Examinees were 30–80 years old and free of cancer in the baseline years of 1996 and 1997. An independent population of 547,874 men and 415,875 women who participated in 1998 and 1999 examinations was used to validate the model. Model validation was done by evaluating its performance in terms of discrimination and calibration ability using the C-statistic and Hosmer-Lemeshow-type chi-square statistics.

**Results:**

Age, body mass index, serum cholesterol, family history of cancer, and alcohol consumption were included in all models for men, whereas age, height, and meat intake frequency were included in all models for women. Models showed moderately good discrimination ability with *C*-statistics between 0.69 and 0.78. The C-statistics were generally higher in the models for men, whereas the calibration abilities were generally better in the models for women.

**Conclusions:**

Colorectal cancer risk prediction models were developed from large-scale, population-based data. Those models can be used for identifying high risk groups and developing preventive intervention strategies for colorectal cancer.

## Introduction

Colorectal cancer is one of the most rapidly increasing cancer in the Korean population, with annual percent changes of 6.2% in men and 6.8% in women between 1999 and 2009 [1]. Although the mortality rate from colorectal cancer started to decline in younger generations and women [2], colorectal cancer is still ranked the fourth most common cause of cancer death [3].

Several risk prediction models for colorectal cancer have been developed and validated in different populations [4]–[11]. The major roles of risk prediction models are: 1) to identify individuals at high risk of developing the disease who can then be offered individually tailored clinical management, targeted screening and interventions to reduce the burden of disease and 2) to identify new risk factors for the disease through research [11].

Recent literature suggests that the distribution of molecular subtypes of colorectal cancer differ by subsites [12], [13]. In our previous study, we reported that risk factor profiles differed by sex, and by the anatomical locations of the colorectal cancer [14]. Therefore, the focus of the present study was to develop colorectal cancer risk prediction models for overall colorectal cancer, proximal colon cancer, distal colon cancer, and rectal cancer for the Korean population; utilizing a large set of health examination data.

## Methods

### Study population

This study was approved by the Institutional Review Board of the National Cancer Center, Korea (IRB no. NCCNCS 09-305). The need for participants' consent was waived by the ethics committee because this study involved routinely collected medical data that were anonymously managed in all stages, including data cleaning and statistical analyses.

Two independent sets of population were incorporated into this study. The first data set was used for model development, which consisted of men and women who participated in a medical examinations provided by the National Health Insurance Corporation (NHIC) between 1996 and 1997. Details of the study design have been described elsewhere [14]. Participants were asked to fill out self-administered questionnaires on alcohol consumption, cigarette smoking habits, regular exercise, family history of cancer, dietary preferences, and frequency of meat consumption. Additional information about female reproductive factors (i.e., age at menarche, age at first childbirth, menopausal status, and age at menopause) were also collected. Height and weight were measured directly, and body mass index (BMI) was calculated as the weight in kilograms divided by the height in meters squared.

The second data set was used for model validation, which consisted of those participated in a medical examinations in 1998 and 1999. Those who were included in the final analysis were between 30 and 80 years old, without previous history of cancer, and with no missing information for any of the major risk factor variables (i.e., height, weight, fasting serum glucose, total serum cholesterol, family history of cancer, cigarette smoking status (current/ex-/non-smokers), and alcohol consumption frequency). The number of study subjects included were 1,326,058 (846,559 men and 479,499 women) for development set, and 963,749 (547,874 men and 415,875 women) for validation set.

### Cancer Ascertainment

The incidence of cancer was ascertained from the Korean Central Cancer Registry (KCCR) database, and death information from the Korean National Statistical Office up to December 2007. The subsites of colorectal cancer were categorized by the International Classification of Disease 10th edition (ICD-10) code as follows: proximal colon (C180–C185), distal colon (C186–C187), and rectum (C19–C20). Cancers with an overlapping lesion of the colon (C188), and those were not otherwise specified (C189) were excluded from the sub-site analysis.

### Statistical analysis

Five models were developed for overall colorectal cancer, colon cancer, right colon cancer, left colon cancer, and rectal cancer, separately for men and women. The Cox proportional-hazard regression models were used for developing prediction equations in development set. Colorectal cancer occurrences were counted as an event on the date of hospital admission recorded in the Cancer Registration data. Subjects were censored at the date of death ascertained from the death certificate database, or on the end date after eight years of follow-up.

Crude and age-adjusted analyses were performed for each risk factor. Age and the quadratic terms of age were centralized by subtracting the mean age of the study participants. The risk factors considered for the models were age, age-squared, height, BMI, family history for cancer, fasting glucose, serum cholesterol, cigarette smoking habit, alcohol intake, and meat consumption frequency. All of the risk factors except age were included as categorical variables in the model. BMI was categorized according to the WHO criteria for the Asian population (<25.0 *vs.* ≥25.0). Height was divided into quartiles and the first quartile was used as the reference. Variable selection (forward, backward and stepwise) methods with selection and exclusion criteria of type I error 0.15 were considered in the multivariate model to build the risk prediction model.

The baseline survival estimate for the mean values of the risk factors for time t (t = 5 years) was estimated by the following equation:
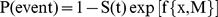
where 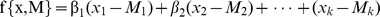.

Here, 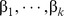 are the regression coefficient estimates, 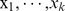 are the risk factors for each individual and 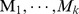 are the mean values for each risk factor in the study population. S(t) is the baseline survival estimate at time t (t = 5 years) when all the risk factors are at their mean values.

Discrimination was quantified by calculating the C-statistic for the survival model [15]. The C-statistic is a concordance measure analogous to the Receiver Operating Characteristic (ROC) Curve area for the logistic model [16]. The value indicates the probability that a model produces higher risk for those who develop breast cancer within five years of follow-up, compared with those who do not develop colorectal cancer [16].

A Hosmer-Lemeshow (H-L) type 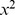 statistic was used for calibration [15]. The 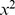 statistic was calculated by first dividing the data into 10 groups (deciles) by ascending order of predicted probabilities produced by the model. Then, in each decile, the average predicted probabilities were compared to the actual event rate estimated by the Kaplan-Meier approach. Values exceeding 20 can be considered a significant lack of calibration [17].

In addition, the expected (E) and the observed (O) numbers of cancer cases were compared for overall colorectal cancer, and each subsites. All statistical analyses were performed using SAS version 9.1 (SAS institute, Cary, NC).

## Results

During the follow-up period, 6,492 men and 2,655 women were developed colorectal cancer in the development set. Among the men, there were 1,143 proximal colon cancers, 1,725 distal colon cancers, and 3,146 rectal cancers. Among the women, there were 604 proximal colon cancers, 606 distal colon cancers, and 1,252 rectal cancers. Cases with overlapping lesions in the colon or whose cancers were not otherwise specified lesions were excluded (478 men and 193 women).

In validation, 3,555 men and 1,969 women were diagnosed with colorectal cancer. Among the men there were 605 proximal colon cancers, 909 distal colon cancers, and 1,764 rectal cancers. Among the women, there were 433 proximal colon cancers, 448 distal colon cancers, and 958 rectal cancers.

### The risk factors included in the models

The risk factors included in the risk prediction models were listed in Table 1 (men) and Table 2 (women). Age, height, family history for cancer, and amount of alcohol consumed were included in all models for men. Body mass index was included in all models except for the one for right colon cancer.

**Table 1:** Relative risks (RR) and 95% confidence intervals (CIs) of variables used for the risk prediction models for **male colorectal cancer**: National Health Insurance Corporation Study, Korea.

Risk factor category	Colorectum (C18–C20)	Colon (C18)	Right colon	Left colon	Rectum (C19–C20)
	HR (95% CI)	HR (95% CI)	HR (95% CI)	HR (95% CI)	HR (95% CI)
Age-Mean_age_, years	1.11 (1.11, 1.12)	1.12 (1.11, 1.12)	1.10 (1.09, 1.11)	1.13 (1.12, 1.14)	1.11 (1.10, 1.11)
(Age-Mean_age_)^2^, years^2^	1.00 (1.00, 1.00)	1.00 (1.00, 1.00)	1.00 (1.00, 1.00)	1.00 (1.00, 1.00)	1.00 (1.00, 1.00)
Height (cm)					
≤165	1.00 (reference)	1.00 (reference)	-	1.00 (reference)	1.00 (reference)
>165, ≤168	1.05 (0.98, 1.13)	1.08 (0.97, 1.19)	-	1.16 (1.01, 1.32)	1.05 (0.95, 1.16)
>168, ≤172	1.17 (1.10, 1.25)	1.21 (1.10, 1.34)	-	1.28 (1.13, 1.44)	1.14 (1.04, 1.25)
>172	1.21 (1.13, 1.30)	1.26 (1.13, 1.40)	-	1.38 (1.20, 1.58)	1.16 (1.05, 1.29)
BMI (kg/m^2^)					
<25.0	1.00 (reference)	1.00 (reference)	1.00 (reference)	1.00 (reference)	1.00 (reference)
≥25.0	1.13 (1.07, 1.19)	1.20 (1.11, 1.30)	1.11 (0.98, 1.26)	1.27 (1.15, 1.41)	1.07 (0.99, 1.15)
Glucose (mg/dL)					
<126	1.00 (reference)	-	-	-	1.00 (reference)
≥126	1.10 (1.01, 1.20)	-	-	-	1.18 (1.05, 1.33)
Cholesterol (mg/dL)					
≤200	1.00 (reference)	1.00 (reference)	-	1.00 (reference)	1.00 (reference)
201–239	1.10 (1.04, 1.16)	1.10 (1.01, 1.19)	-	1.09 (0.98, 1.21)	1.14 (1.06, 1.24)
≥240	1.16 (1.08, 1.26)	1.09 (0.97, 1.23)	-	1.20 (1.03, 1.39)	1.25 (1.12, 1.40)
Family history of cancer					
No	1.00 (reference)	1.00 (reference)	1.00 (reference)	1.00 (reference)	1.00 (reference)
Yes	1.22 (1.14, 1.29)	1.31 (1.19, 1.43)	1.29 (1.11, 1.48)	1.33 (1.18, 1.49)	1.11 (1.02, 1.22)
Alcohol consumption (g/day)					
0	1.00 (reference)	1.00 (reference)	1.00 (reference)	1.00 (reference)	1.00 (reference)
1–14.9	1.10 (1.03, 1.18)	1.18 (1.07, 1.30)	1.14 (0.98, 1.33)	1.21 (1.07, 1.37)	1.05 (0.96, 1.16)
15–24.9	1.21 (1.13, 1.31)	1.30 (1.16, 1.45)	1.20 (1.00, 1.43)	1.37 (1.19, 1.58)	1.16 (1.04, 1.29)
25 or more	1.26 (1.18, 1.35)	1.31 (1.19, 1.45)	1.24 (1.06, 1.45)	1.38 (1.21, 1.56)	1.22 (1.11, 1.34)
Meat consumption (1 week)					
≤1 time	1.00 (recference)	1.00 (reference)	1.00 (reference)	-	-
2–3 times	1.04 (0.98, 1.09)	1.04 (0.97, 1.13)	1.09 (0.97, 1.23)	-	-
≥4 times	1.15 (1.04, 1.27)	1.17 (1.00, 1.35)	1.23 (0.98, 1.55)	-	-

**Table 2:** Relative risks (RR) and 95% confidence intervals (CIs) of variables used for the risk prediction models for **female colorectal cancer**: National Health Insurance Corporation Study, Korea.

Risk factor category	Colorectum (C18–C20)	Colon (C18)	Right colon	Left colon	Rectum (C19–C20)
	HR (95% CI)	HR (95% CI)	HR (95% CI)	HR (95% CI)	HR (95% CI)
Age-Mean_age_, years	1.08 (1.07, 1.09)	1.08 (1.08, 1.09)	1.10 (1.08, 1.11)	1.07 (1.06, 1.08)	1.08 (1.07, 1.08)
(Age-Mean_age_)^2^, years^2^	1.00 (1.00, 1.00)	1.00 (1.00, 1.00)	1.00 (1.00, 1.00)	1.00 (1.00, 1.00)	1.00 (1.00, 1.00)
Height (cm)					
≤151	1.00 (reference)	1.00 (reference)	1.00 (reference)	1.00 (reference)	1.00 (reference)
>151, ≤155	1.16 (1.05, 1.28)	1.31 (1.13, 1.52)	1.36 (1.11, 1.66)	1.27 (1.03, 1.58)	1.06 (0.91, 1.23)
>155, ≤158	1.16 (1.04, 1.31)	1.20 (1.01, 1.42)	1.26 (0.99, 1.61)	1.14 (0.89, 1.46)	1.15 (0.97, 1.36)
>158	1.22 (1.09, 1.37)	1.24 (1.04, 1.47)	1.04 (0.80, 1.36)	1.41 (1.12, 1.79)	1.23 (1.04, 1.45)
BMI (kg/m^2^)					
<25.0	-	-	1.00 (reference)	-	-
≥25.0	-	-	1.16 (0.98, 1.37)	-	-
Glucose (mg/dL)					
<126	1.00 (reference)	1.00 (reference)	1.00 (reference)	1.00 (reference)	-
≥126	1.21 (1.05, 1.40)	1.29 (1.05, 1.58)	1.28 (0.97, 1.70)	1.27 (0.94, 1.72)	-
Family history of cancer					
No	1.00 (reference)	1.00 (reference)	1.00 (reference)	1.00 (reference)	-
Yes	1.18 (1.07, 1.29)	1.29 (1.12, 1.48)	1.18 (0.97, 1.44)	1.40 (1.16, 1.68)	-
Alcohol consumption (g/day)					
0	-	-	-	-	1.00 (reference)
1–14.9	-	-	-	-	1.00 (0.83, 1.20)
15 or more	-	-	-	-	1.48 (1.10, 1.99)
Meat consumption (1 week)					
≤1 time	1.00 (reference)	1.00 (reference)	-	1.00 (reference)	1.00 (reference)
2–3 times	1.07 (0.99, 1.16)	1.14 (1.01, 1.29)	-	1.16 (0.98, 1.38)	1.03 (0.91, 1.16)
≥4 times	1.29 (1.12, 1.49)	1.26 (1.02, 1.56)	-	1.34 (0.99, 1.81)	1.39 (1.14, 1.70)

In women, age and height were included in all models. Fasting glucose and family history of cancer were included in all models except that for rectal cancer, and meat consumption frequency was included in all models except that for the right colon. BMI was included in the model for right colon only, and frequency of alcohol consumption was included in the model for rectal cancer only.

### Model performance

#### Discrimination

The discriminatory ability of the model was measured using the C-statistic in both development and validation sets (Table 3). The C-statistics for models for men ranged 0.762∼0.786 and those statistics for models for women were 0.678∼0.763. Models for colorectum (0.762 for development set and 0.779 for validation set), left colon (0.786 for development set and 0.779 for validation set), as well as rectum (0.753 for development set and 0.779 for validation set) showed the highest C-statistics in men, whereas models for right colon showed the highest values in women (0.745 for development set and 0.763 for validation set).

**Table 3:** C statistic and Hosmer-Lemeshow type chi-square test for colorectal cancer risk prediction models for development set (8-year risk) and validation set (5-year risk).

Models	Colorectum	Right colon	Left colon	Colon	Rectum
Male					
Development set					
C (95% CI)	0.762 (0.755, 0.769)	0.740 (0.721, 0.759)	0.786 (0.772, 0.799)	0.767 (0.756, 0.778)	0.753 (0.743, 0.763)
Chi-square value (p-value)	14.567 (0.1035)	7.162 (0.6203)	8.567 (0.4782)	11.871 (0.2207)	10.788 (0.2906)
Validation set					
C (95% CI)	0.779 (0.768, 0.789)	0.762 (0.734, 0.789)	0.779 (0.758, 0.801)	0.770 (0.753, 0.787)	0.779 (0.766, 0.793)
Chi-square value (p-value)	31.383 (0.0003)	24.540 (0.0035)	8.189 (0.515)	18.575 (0.029)	30.970 (0.0003)
Female					
Development set					
C (95% CI)	0.706 (0.695, 0.718)	0.745 (0.722, 0.768)	0.678 (0.652, 0.704)	0.711 (0.693, 0.728)	0.698 (0.682, 0.714)
Chi-square value (p-value)	8.219 (0.5123)	6.720 (0.6663)	5.936 (0.7463)	6.054 (0.7345)	15.246 (0.0844)
Validation set					
C (95% CI)	0.726 (0.712, 0.741)	0.763 (0.736, 0.791)	0.690 (0.659, 0.721)	0.723 (0.702, 0.743)	0.716 (0.696, 0.737)
Chi-square value (p-value)	13.129 (0.1569)	4.597 (0.8679)	10.415 (0.3180)	8.421 (0.4924)	12.279 (0.1980)

#### Calibration

**Figure 1:**
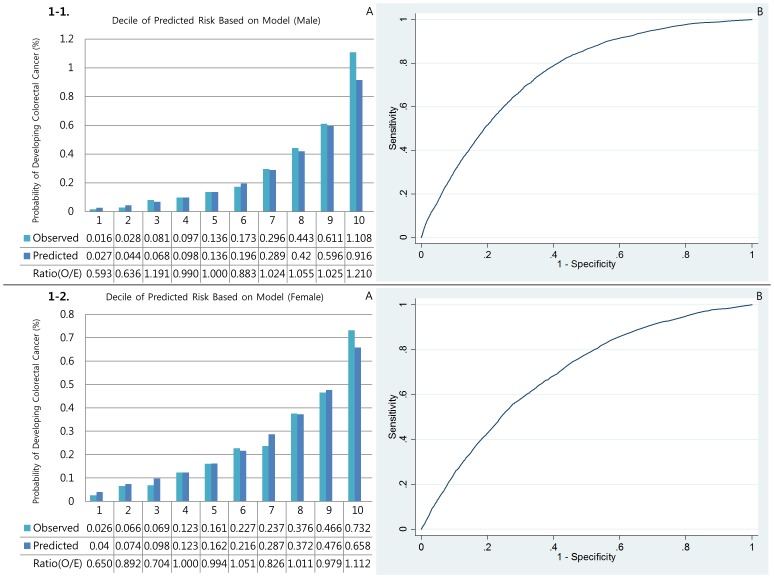
Discrimination (A) and calibration (B) of the Colorectal cancer prediction models.

**Figure 2:**
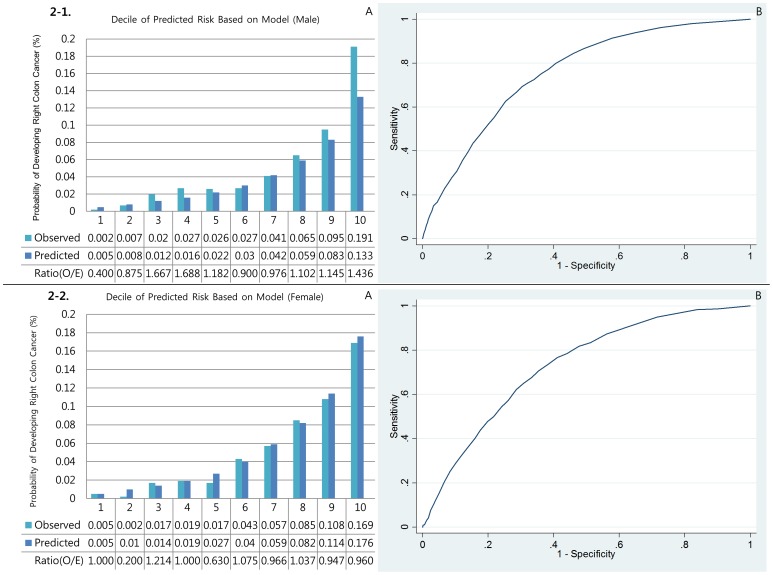
Discrimination (A) and calibration (B) of the Right colon cancer prediction models.

**Figure 3:**
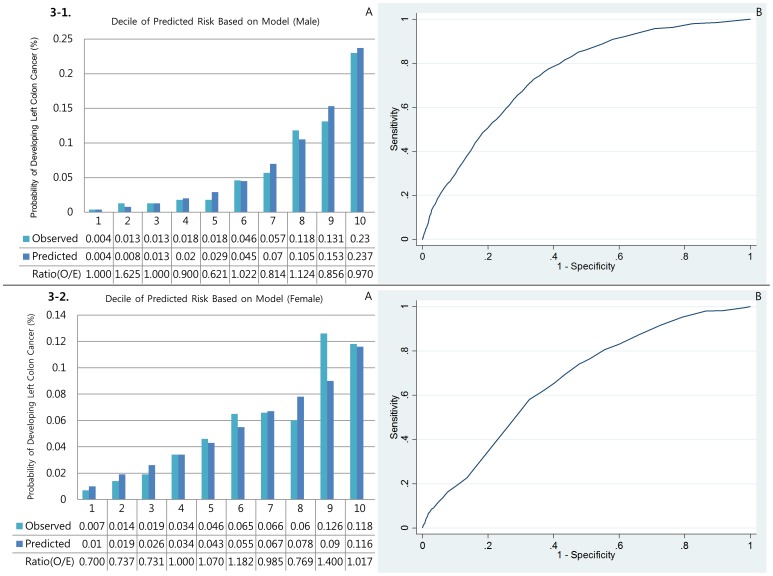
Discrimination (A) and calibration (B) of the Left colon cancer prediction models.

**Figure 4:**
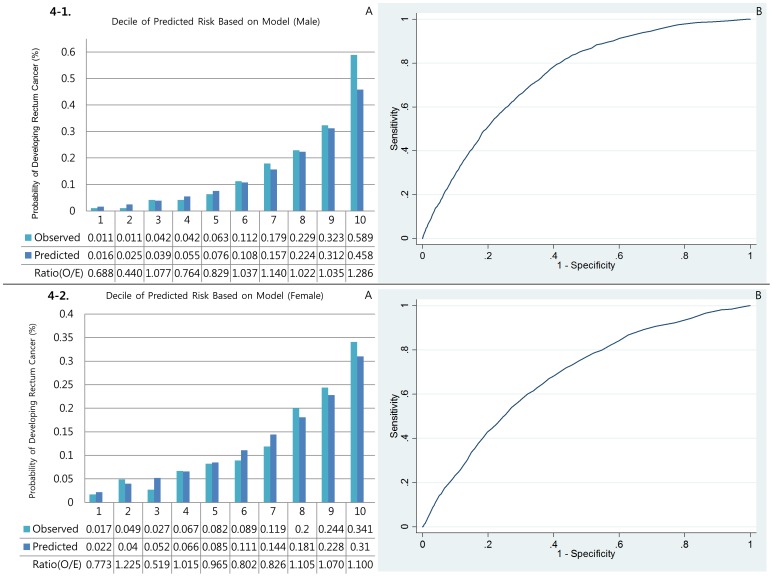
Discrimination (A) and calibration (B) of the Rectum cancer prediction models.

**Figure 5:**
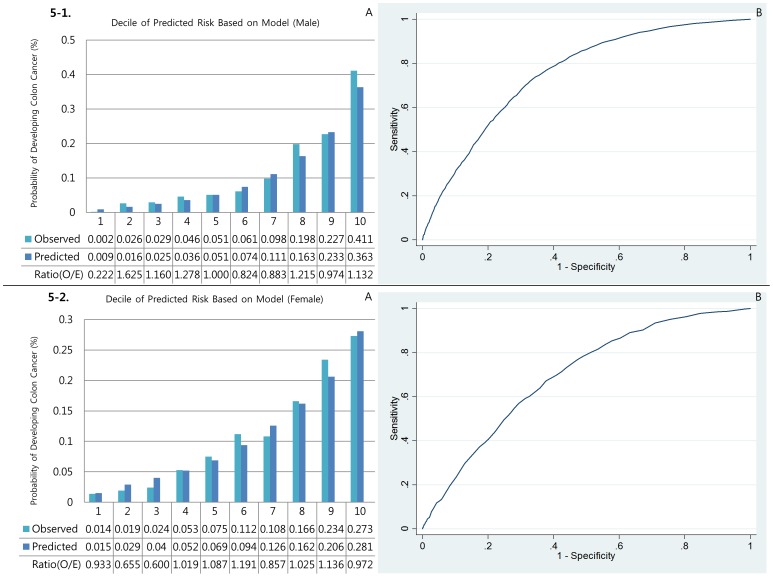
Discrimination (A) and calibration (B) of the Colon cancer prediction models.

Figures 1-A, 2-A, 3-A, 4-A, and 5-A show the calibration plots for the overall colorectal cancer model as well as E/O ratios of validation sets for male colorectal, right colon, left colon, rectal, and colon cancers, respectively, and figures 1-B, 2-B, 3-B, 4-B, and 5-B show those for female, respectively. Table 3 presented the Hosmer-Lemeshow-type chi-square values. In general, the event rates predicted by the models were very close to the actual event rates in male models. Only models for left colon cancer in men did not show significant prediction power. In women, however, none of the models showed significant prediction ability.

## Discussion

Recent epidemiological and clinical information suggest that colon cancer and rectal cancer are distinct diseases [12], [13], [18]. In addition, proximal and distal colons are different in embryologic origins, morphologic appearance of mucosa, physiological function, and bile acid composition [19], [20]. Among several colorectal cancer risk prediction models developed and validated [11], only one study provides separate models for proximal and distal colon, and rectum [6]. One study provided separate models for colon cancer and rectal cancer [7]. Previously, we published an article on the risk factor profiles for different colorectal cancer subsites [14]. The prediction models were developed using the same dataset for the model development with a longer follow-up period. In addition, an independent population was used for model validation.

The models showed moderately good discrimination ability. The model for overall colorectal cancer showed the best calibration ability. Among the models for women, that for right colon cancer showed the highest discrimination ability and that for left colon cancer showed the lowest C-statistics. Unfortunately none of the models showed any meaningful calibration ability. Still, our models showed C-statistics that were comparable with, or even higher than, other colorectal cancer risk prediction models [11]. The C-statistics for three previous models 0.67–0.71 for Harvard Cancer Risk Index, 0.61 for the US study, and 0.62–0.66 for Japanese study, respectively, whereas those for our models were 0.68–0.78 [11]. Indeed, model for left colon cancer in women did not reach C-statistics of 0.7. Two studies provided calibration statistics as ratio of observed vs. expected colorectal cancer events (O/E) [7], [8]. The O/E ratios varied depend on risk factor profile [7], [8]. In a Japanese model for men, the Hosmer-Lemeshow chi-square p-value was 0.08 [7].

The incidence rate for colorectal cancer in women is two thirds that in men for the Korean population [21]. Relatively low cancer incidence rates for women, compared to men, may restrict the statistical power of models for women. Lack of detailed information about female-specific risk factors such as reproductive and hormonal factors may be another reason for the limited power of calibration [22].

The current risk prediction models aim to assess the probability of sporadic colorectal cancer risk. Hereditary colorectal cancer syndromes such as hereditary nonpolyposis colorectal cancer and familial adenomatous polyposis are known to account for up to 2% of overall colorectal cancers [23], [24]. Mixing hereditary cancer cases into our study cohort may have diluted the relative risks due to environmental factors.

The strengths of the current study include a large sample size and completeness of cancer follow-up by data linkage to cancer registration and death certificates. Limitations include limited information on dietary risk or protective factors such as calcium and fiber intake [25], or non-dietary factors such as nonsteroidal anti-inflammatory drugs [26]. Previous colonoscopy which may reduce the incidence of cancer was not considered in the model.

In conclusion, risk prediction models for colorectal cancer developed by utilizing large insurance-based data sets from the Korean population, show reasonable discrimination ability. These models help define groups at high risk for colorectal cancer and help guide them to change risk behaviors as well as to undergo cancer screening.
